# Unlocking Deformation Path in Asymmetric Rolling by Texture Simulation

**DOI:** 10.3390/ma13010101

**Published:** 2019-12-24

**Authors:** Satyaveer Singh Dhinwal, Laszlo S Toth

**Affiliations:** 1 Laboratory of Excellence on Design of Alloy Metals for Low-Mass Structure (Labex-DAMAS), Université de Lorraine, 57070 Metz, France; laszlo.toth@univ-lorraine.fr; 2 Université de Lorraine, CNRS, Arts et Métiers ParisTech, LEM3, F-57000 Metz, France

**Keywords:** asymmetric rolling, deformation texture, VPSC modeling, strain path, extra-low carbon steel

## Abstract

The texture evolution is wearing the signature of the deformation path in plastic deformation. In asymmetric rolling, plain strain compression and shear are the main components of the imposed strain. In this work, viscoplastic self-consistent (VPSC) simulations of the texture evolution were used to determine the combination and sequence of the two deformation components. It has been found that the deformation path is composed of two parts in asymmetric rolling: it is first essentially rolling, followed by the simple shear process. Simultaneous rolling and shear process cannot produce the observed textures, while the decomposed simulation can reproduce it faithfully.

## 1. Introduction

Asymmetric rolling is considered as the most viable method to introduce through thickness shear deformation. As an example of industrial interest in asymmetric rolling, electrical (Fe-Si) steel manufacturing benefits when the “Goss” {110} <001> texture component is strengthened in ferritic steels through the whole thickness when the sheet is rolled in asymmetric conditions [1]. The strengthening of the “Goss” texture component is vital for electrical steels in order to minimize the Eddy current loss, which increases the efficiency of transformers. Through thickness, shear deformation in rolling can be introduced in three ways: by different friction conditions on the two surfaces of the sheet, differences in roll diameters, or by different angular speeds of the two rolls. The benefit of induced extra shear deformation has also been reported to increase grain fragmentation and to speed up recrystallization kinetics in postannealing treatment [2,3,4,5]. Depending on the imposed rolling parameters in asymmetric rolling, the resultant texture is between plane-strain compression and simple shear texture. It is also evidenced in a recent work [1] that most texture components of symmetric rolling appear as shear-texture components by simply rotating the rolling texture 30–35° around the transverse direction of the sheet. Rotation of the texture is possible due to the shear component, which involves high rigid-body rotation for the texture. Such rotations of the texture have been observed in several works [6,7]. The highest rotations can be obtained when the thickness reduction is very high in a given pass during asymmetric rolling [1,2]. Because the shear component induces the rotations, it is important to know its value and also its articulation with the rolling strain. In our recent experimental work, inserts were used to obtain the shear value experimentally [2]. In the present work, we propose an analytical model that can be readily used for tailoring the asymmetric rolling process for the best shear texture. An extra low carbon grade ferritic steel was considered as a reference material in the simulations.

## 2. Material and Methods

**Figure 1:**
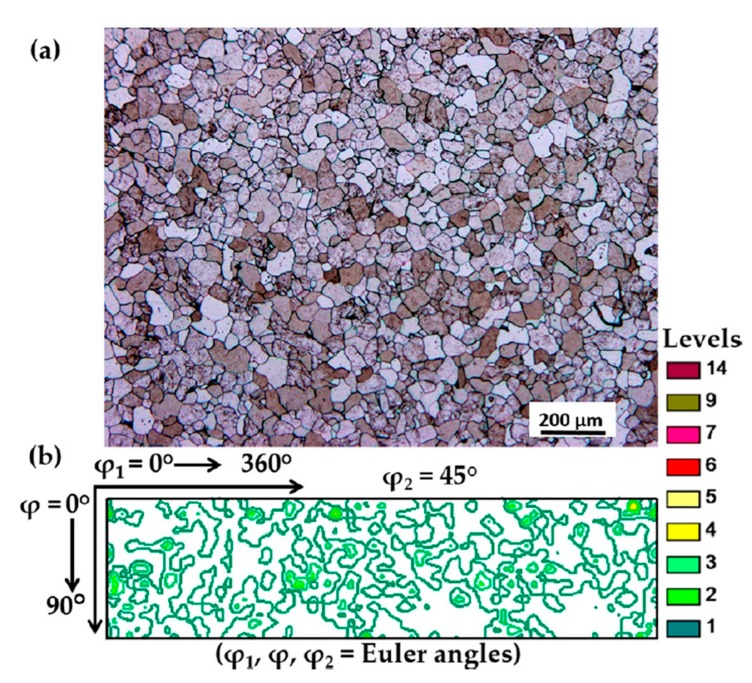
(**a**) The microstructure after homogenization. (**b**) The φ_2_ = 45° orientation distribution function (ODF) section of the initial texture for the extra low carbon steel that was examined.

Figure 1 represents the initial microstructure and texture of our homogenized extra-low carbon steel used for the rolling experiments. The nominal composition is given in Table 1. Detailed information about the rolling parameters and experimental investigation can be found in [2].

The viscoplastic self-consistent (VPSC) approach was employed for the texture simulations. The VPSC approach is a well-known polycrystal code that is based on the interaction of an inclusion (a grain) with the whole polycrystal represented by a homogeneous medium. More details about the VPSC approach can be found in [8,9].

**Figure 2:**
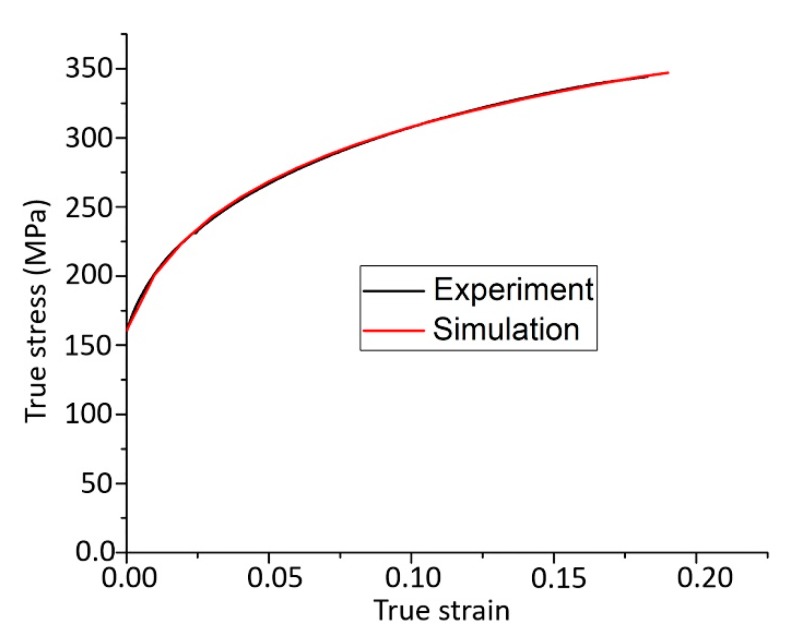
Measured and simulated strain hardening curves for tensile testing of extra-low carbon steel at room temperature.

The experimental initial texture was discretized to 10,000 grain orientations using ATEX software (version 1.55, ATEX-Université de Lorraine, Metz, France) [10]. The initial grain shapes were quite equiaxed, so they were assumed initially to be spherical. Their shapes were allowed to develop during the simulation according to the applied strain path. The 12 {110} <111> and 12 {112} <111> slip system families were considered for deformation, with initial shear strengths of 83 and 79 MPa, respectively. Hardening was modeled using the approach developed by Kalidindi et al. [11,12] and Zhou et al. [13]. The hardening parameters were obtained from another VPSC simulation, by nearly perfectly reproducing the strain-hardening curve measured in tensile testing on the same material. Figure 2 shows the measured and simulated hardening curves. The fit is so perfect that they can be hardly distinguished. The parameters of the strain-hardening approach appear in Equations (1) and (2) [11,12,13]:

(1) $${\dot{\tau }}_{0}^{\left(i\right)}=\sum_{j=1}^{N}H^{ij}\left|{\dot{\gamma }}^{j}\right|,\;\mathrm{where}\;i,\;j=1\ldots N.$$

Here, $\tau_{0}^{\left(i\right)}$ is the strength of the slip system indexed by *i*, ${\dot{\gamma }}^{j}$ is the slip rate in slip system *j*, *N* is the total number of slip systems, and H^*ij*^ is the self- and latent-hardening matrix (dimension *N* × *N*). The latter is defined by:

(2) $$H^{ij}=q^{ij}h_{0}{\left(1-\frac{\tau_{0}^{i}}{\tau_{sat}}\right)}^{a}$$

where *h*_0_ and *a* are hardening parameters and *τ_sat_* is the saturation stress. *q^ij^* is a symmetric-hardening matrix which expresses the interaction between slip systems. The elements of this hardening matrix were determined according to four possible geometrical configurations of the *i* and *j* slip systems: coplanar slip (*q*_1_), collinear slip (*q*_2_), perpendicular slip (*q*_3_), and for all other configurations, the same coefficient (*q*_4_) was allocated. By a fitting process, the following hardening parameter values were identified for extra-low carbon steel: *h*_0_ = 3980 MPa, *a =* 24, *τ_sat_* = 1080 MPa, *q*_1_ = 1.0, *q*_2_ = 1.5, *q*_3_ = 2.0, *q*_4_ = 1.5. As these parameters are intrinsic to the material, they can be employed not only for tension testing but also for rolling.

## 3. Results and Discussion

The superposition of rolling and shear can be expressed by a single parameter *p* in the velocity gradient tensor (*L*) of the asymmetric rolling process [6,7]:

(3) $$L=\left|\dot{\varepsilon }\right|\left(\begin{array}{ccc} 1 & 0 & p\\ 0 & 0 & 0\\ 0 & 0 & -1 \end{array}\right)$$

Here, the rolling plane is perpendicular to axis 3 and rolling direction is axis 1, $\dot{\varepsilon }$ is the strain rate in thickness reduction, and *p* is the ratio between the shear and compression strain rates:

(4) $$p=\dot{\gamma }/\left|\dot{\varepsilon }\right|$$

If a vertical insert is placed into the sheet before testing, parallel to the sheet normal, and *p* is constant during rolling (i.e., the strain path is proportional), *p* can be calculated by the following formula [6]:

(5) $$p=\frac{h_{f}}{h_{i}-h_{f}}\tan\left(\alpha_{f}\right),$$

where $\alpha_{f}$ is the final orientation of the insert with respect to the sheet normal after rolling and $h_{i}$ and $h_{f}$ are the thicknesses of the sheet before and after rolling, respectively. Under these conditions, the total imparted shear $\gamma_{f}$ can be obtained from Equation (4) by integration and using Equation (5):

(6) $$\gamma_{f}=\frac{h_{f}}{h_{i}-h_{f}}\tan\left(\alpha_{f}\right)\ln\left(\frac{h_{i}}{h_{f}}\right).$$

Table 2 shows the numerical values for the insert angles, rolling strain, shear strain $\gamma_{f}$, and *p* values for four thickness reduction values, which we carried out in asymmetric rolling of extra-low carbon steel, using a roll-diameter ratio of 2.

In earlier investigations, very high *p*-values (p≫1) were proposed from texture simulations to obtain shear texture during asymmetric rolling [7,14]. However, a recent experimental investigation points out that substantial rotation of the rolling texture towards shear texture can also occur for lower *p*-values if the reduction in thickness is at least 50–57% in one pass, and the roll diameter ratio is above 1:1.6 [1]. This might be due to a non-constant value of the *p* parameter during asymmetric rolling, which is the subject of the present work. More precisely, instead of superimposing rolling and shear simultaneously, it is proposed here that the strain path during asymmetric rolling can be decomposed into two main parts: first rolling with a small amount of shear, then finishing with only simple shear. This simple decomposition of the strain path can be convincingly justified by texture simulations, which are presented next.

**Figure 3:**
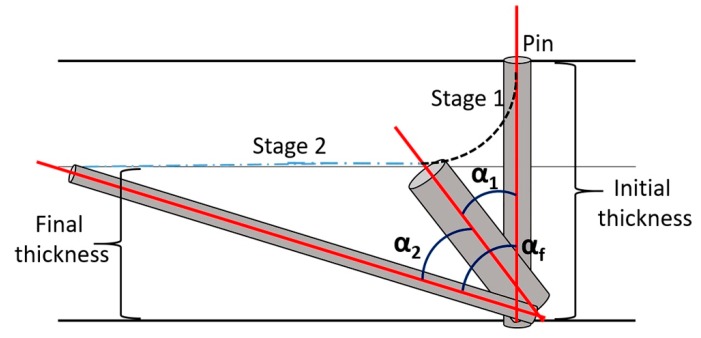
Schematic of deformation path followed by a vertically inserted pin in asymmetric rolling.

Simulations were carried out for all four deformation conditions shown in Table 2. In order to save space, here we show results only for the smallest (30%) and largest thickness reductions (65%). In both cases, three simulations were done. In the first condition (Case 1), the strain path was split into two parts; first rolling with shear, where the shear strain was only 20% of the total value, followed by only simple shear by the remaining 80%, in a second step. In Case 2, rolling and shear were fully decoupled; only rolling was imposed in the first strain path, and simple shear in the second path. For these simulations, the amount of shear was obtained from the orientation of the inserted pin in the experiment (Table 2). Namely, by taking into account that no shear was assumed (in the second case), the orientation of the pin could only change once the thickness reduction was achieved, so the shear value is equal to the tangent of the orientation of the pin with respect to the sheet normal (Figure 3). This value was also assumed in the first case, where 20% shear was added to the rolling in the first phase of the strain path. These shear values are presented in Table 2. In Case 3, both the shear and rolling strain components were imposed simultaneously in a single simulation for a proportional strain path defined by the velocity gradient tensor (Equation (3)) and Equations (4)–(6).

**Figure 4:**
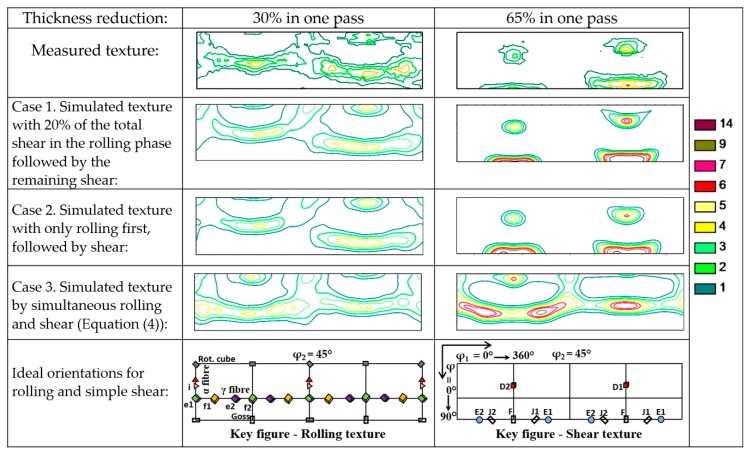
Measured and simulated textures in low carbon steel in asymmetric rolling for a roll-diameter ratio of 2 at room temperature, in single passes, after 30% and 65% thickness reductions, presented in ODF form for the φ_2_ = 45° section of Euler space.

All textures are presented for the φ_2_ = 45° section of the Euler orientation space shown in Figure 4, which contains all ideal orientations of bcc (Body Centered Cubic) rolling and shear textures. By comparing the simulated textures with the experimental ones, it is apparent that the experiment is very well reproduced for the first and second cases, when the strain path was split so that the major part of the shear component was applied in the second phase of asymmetric rolling. Indeed, all components are reproduced in the correct positions and nearly with the same intensities as in the experiment. The agreement is slightly better when 20% of the total shear was added to the first phase of rolling. On the contrary, by simultaneous rolling and shear (Case 3), the simulation results are not satisfactory.

**Figure 5:**
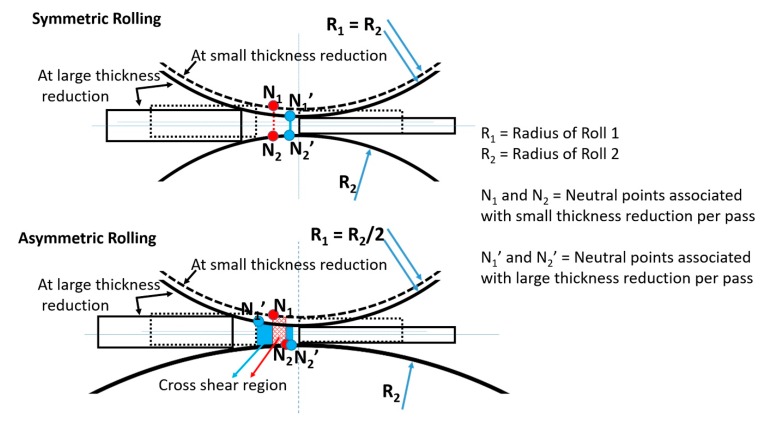
Comparative schematic that shows the positions of the neutral points in symmetric and asymmetric rolling for small and high thickness reductions, per pass. The cross-shear regions for asymmetric rolling are shown in blue and red.

The texture simulation results strongly support the idea that in asymmetric rolling the material first undergoes mostly rolling strain, then, in a second phase, only shear strain is applied. The origin of this sequence of strain modes can be understood by examining the positions of the neutral points. They are located at the point where the velocities of the roll and the sheet are equal. It is also the point where the friction force changes its directional sense. Figure 5 shows schematically the positions of the neutral points for symmetric and asymmetric rolling, for low and high reduction per pass. For symmetric rolling, the neutral points are positioned on the same vertical line on the top and bottom surfaces of the sheet, regardless of the applied thickness reduction. For asymmetric rolling, the surface velocity of the bottom roll is higher, so the bottom neutral point is shifted to the right with respect to the top one. This shift creates a “cross-shear zone” [15,16,17] that is longer for higher thickness reduction. As the cross-shear zone is near to the exit point of rolling, where the material flow is tangent to the rolling direction, the deformation state is close to simple shear. Before this zone, where the material enters, the thickness reduction is much higher than in the cross-shear zone, so most of the deformation is thickness reduction, while in the second zone, it is closer to simple shear. Therefore, the geometry conditions justify the change of strain mode during asymmetric rolling.

## 4. Conclusions

Texture simulations were successfully used in this work to identify the strain mode in asymmetric rolling, which is essentially composed of two strain modes: the material first experiences mostly rolling strain and then simple shear before exiting. A fine tuning of the strain mode indicates that some shear can take place during the first stage of deformation.

## Figures and Tables

**Table 1:** Nominal chemical composition of the extra-low carbon steel that was examined (weight percentages).

C	Mn	Si	Al	Cr	Ni	Cu	Ti	Fe
0.030	0.15	0.006	0.043	0.020	0.010	0.003	0.001	balance

**Table 2:** Strain values and pin orientations for four experiments in asymmetric rolling of low carbon steel, using a roll-diameter ratio of 1:2.

Thickness Reduction in a Pass	Pin Inclination Angle (α_f_)	Tan(α_f_)	Rolling Strain	Shear Strain (Equation (4))	Shear Coefficient, *p*	Shear in Stage 1	Shear in Stage 2
30%	14° ± 1°	0.25 ± 0.02	0.36	0.20	0.55	0/0.05	0.20/0.25
50%	35° ± 2°	0.70 ± 0.03	0.69	0.48	0.70	0/0.14	0.56/0.70
57%	54° ± 3°	1.38 ± 0.1	0.84	0.87	1.04	0/0.28	1.10/1.38
65%	63° ± 4°	1.96 ± 0.17	1.05	1.11	1.06	0/0.39	1.57/1.96
